# Chrysophanol inhibits of colorectal cancer cell motility and energy metabolism by targeting the KITENIN/ErbB4 oncogenic complex

**DOI:** 10.1186/s12935-024-03434-x

**Published:** 2024-07-20

**Authors:** Mücahit Varlı, Eunae Kim, Songjin Oh, Sultan Pulat, Rui Zhou, Chathurika D. B. Gamage, Barış Gökalsın, Nüzhet Cenk Sesal, Kyung Keun Kim, Man-Jeong Paik, Hangun Kim

**Affiliations:** 1 College of Pharmacy, Sunchon National University, 255 Jungang-ro, Sunchon, Jeonnam 57922 Republic of Korea; 2 College of Pharmacy, Chosun University, 146 Chosundae-gil, Gwangju, 61452 Republic of Korea; 3 Faculty of Arts and Sciences, Department of Biology, Marmara University, Istanbul, Türkiye; 4 Department of Pharmacology, Chonnam National University Medical School, 160 Baekseoro, Gwangju, 61469 Republic of Korea

**Keywords:** Chrysophanol, Anthraquinone, KITENIN, ErbB4, Metastasis, Aerobic glycolysis, Colorectal cancer

## Abstract

**Background:**

Expression of the KITENIN/ErbB4 oncogenic complex is associated with metastasis of colorectal cancer to distant organs and lymph nodes and is linked with poor prognosis and poor survival.

**Methods:**

Here, we used in vitro and in silico methods to test the ability of chrysophanol, a molecule of natural origin, to suppress the progression of colorectal cancer by targeting the KITENIN/ErbB4 complex.

**Results:**

Chrysophanol binds to ErbB4, disrupting the ErbB4/KITENIN complex and causing autophagic degradation of KITENIN. We demonstrated that chrysophanol binds to ErbB4 according to a molecular docking model. Chrysophanol reversed KITENIN-mediated effects on cell motility, aerobic glycolysis, and expression of downstream effector genes. Moreover, under conditions of KITENIN overexpression, chrysophanol suppressed the production of onco-metabolites.

**Conclusion:**

Chrysophanol suppresses oncogenic activities by targeting the KITENIN/ErbB4 complex.

**Graphical Abstract:**

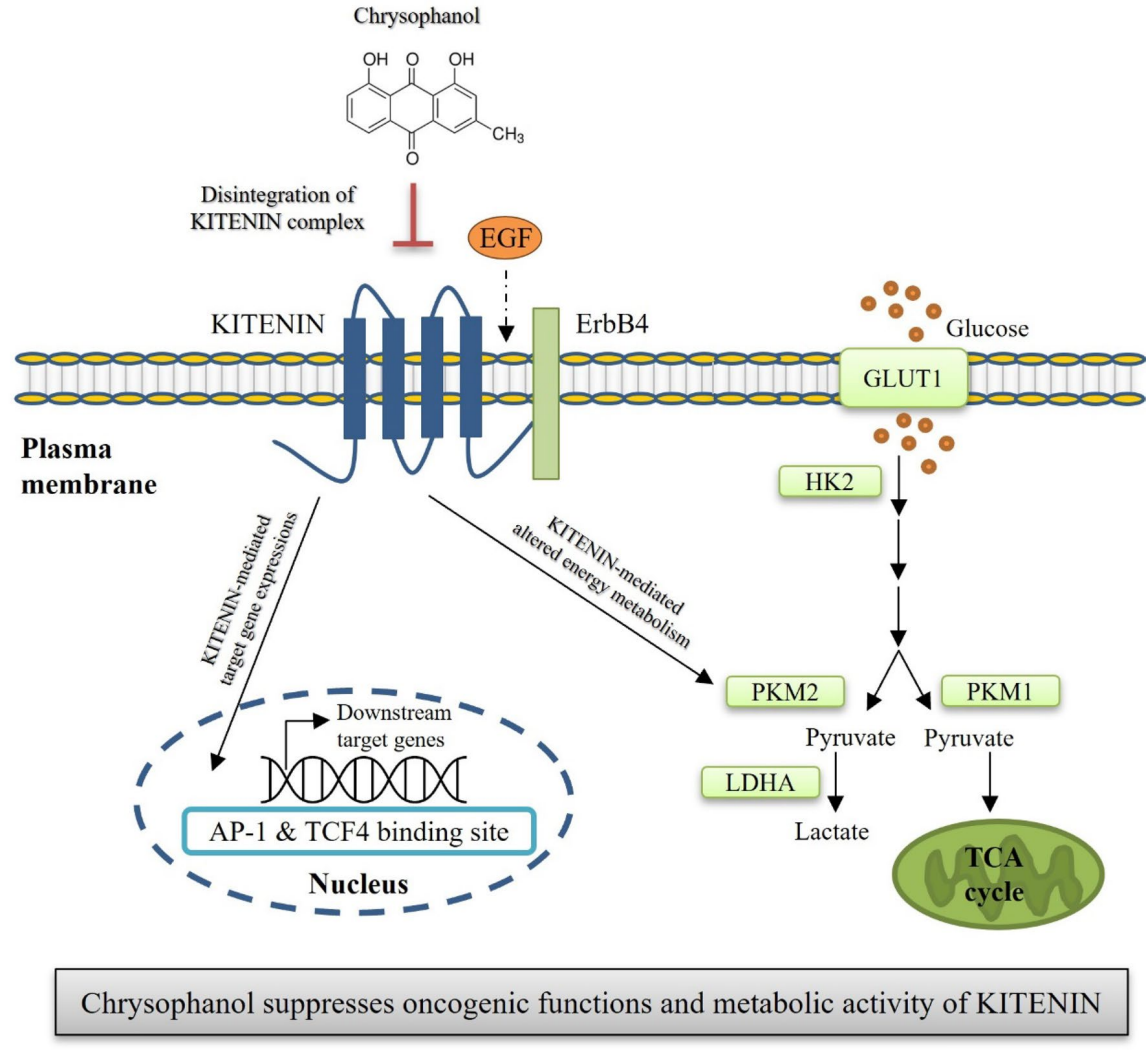

**Supplementary Information:**

The online version contains supplementary material available at 10.1186/s12935-024-03434-x.

## Introduction

Colorectal cancer (CRC) is the second most common cause of cancer death and the third most common type of cancer. The International Agency for Research on Cancer predicts that, by 2040, 3.2 million of CRC patients globally and the mortality rates due to CRC will have increased dramatically [1]. Moreover, 40–50% of CRC patients have metastasis at the time of diagnosis or as recurrent disease upon intended curative therapy. Therefore, robust research focusing on the discovery of new targeted anticancer therapeutics that complement treatment strategies such as surgery, chemotherapy, and radiotherapy is urgently needed [2].

The VANGL1 protein was first identified as having an important role in neural tube formation during embryonic development. Subsequent studies redefined VANGL1 as a metastasis-inducing protein, renaming it KITENIN (KAI1 C-terminal interacting tetraspanin) [3–6]. KITENIN forms a functional complex with ErbB4 and promotes cell invasiveness via the KITENIN/ErbB4–Dvl2–c-jun axis, which is part of the epidermal growth factor (EGF) signaling pathway, independently of EGF receptors. The KITENIN axis also contributes to the development of CRC in settings where there is loss of a functional adenomatous polyposis coli (APC) tumor suppressor gene [6–8]. In addition, CRCs that express functional KITENIN complex develop resistance to the EGF receptor-targeted therapy cetuximab [9]. For these reasons, KITENIN is a promising molecular target for the development of new therapeutics for CRC.

Chrysophanol (1,8-dihydroxy-3-methyl-9,10-anthraquinone) is a naturally-derived molecule belonging to the anthraquinone family that has various biological activities [10]. Anthraquinone compounds have been shown to have anticancer effects, including regulation of cell proliferation, stemness, apoptosis, cell migration and invasion, in several types of cancer [11, 12]. In the current study, we examined whether chrysophanol suppresses KITENIN-mediated cancer progression, and if this effect occurs via disruption of the KITENIN/ErbB4 functional complex.

## Materials and methods

### Cell culture

Human colorectal cancer cell CaCo2, mouse colorectal cancer cell CT26 and human embryonic kidney cell HEK293T were used. CaCo2 cell line modified to empty vector or KITENIN overexpression cells. All cells were cultured in DMEM (GenDepot, Katy, TX, USA) supplemented with 10% fetal bovine serum (FBS) and 1% penicillin–streptomycin solution. 5% CO_2_ in a humidified atmosphere at 37 °C was used for incubation of the cells.

### Cell viability assay

Cells (3 × 10^3^ cells/well) were seeded on 96-well plates, grown overnight, and then treated with 0.78–100 μM concentrations of chrysophanol for 48 h. And then, the cultures were supplemented with MTT. After incubation with MTT at 37 °C for 4 h, the cells were lysed with 150 μL of DMSO. Absorbance at 570 nm was measured using a microplate reader and Gen 5 (2.03.1) software (BioTek, Winooski, VT, USA).

### Invasion assay

Transwell chambers (Corning Costar, Corning, NY, USA) covered with 1% gelatin were used to study cell invasion. 5 × 10^4^ CaCo2/EV and CaCo2/KITENIN cells were plated onto precoated inserts in 100 µL of DMEM containing 0.2% bovine serum albumin (BSA) and treated with 1, 5, or 10 µM chrysophanol or DMSO for 24 h at 37 °C. A culture media containing 0.2% BSA and 10 µg/mL fibronectin (EMD Millipore Corp., Billerica, MA, USA) as a chemoattractant was placed in the chamber's lower compartment. Using a Diff-Quick kit (Sysmex, Kobe, Japan), cells in the chamber were fixed and dyed after 24 h. Under a light microscope, the invaded cells were examined in five randomly chosen fields.

### Reporter assay

HEK293T cells were transfected with the KITENIN expression plasmid and AP-1 reporter plasmid using for reporter assay. All plasmids were initially transfected for 12 h. X-treme GENE 9 DNA transfection reagent (Roche, Werk Penzberg, Germany) was used for transfection. After 12 h transfection, cells were treated with 1, 5 or 10 µM chrysophanol or DMSO for 48 h incubation. After incubation, a Dual-Luciferase^®^ reporter assay system using for analysis (Promega, Madison, WI, USA). To check the transfection efficiency, the Renilla luciferase reporter plasmid (pRL-TK) was used as an internal control.

### Quantitative reverse-transcription PCR (qRT-PCR)

2 × 10^5^ number of cells incubated overnight, after cells treated with chrysophanol (1, 5, and 10 µM) or DMSO for 48 h. Total RNA was isolated from cells using RNAiso Plus (TaKaRa,Otsu, Japan) properly to the producer’s instructions. Total RNA (1 μg) from each treated group was converted to cDNA using an M-MLV reverse transcriptase kit (Invitrogen). SYBR Green reagents (Enzynomics, Seoul, Korea) were utilized to assess the relative expression of genes. The list of primers used in this research is reported in Supplementary Table S1. CFX instrument (Bio-Rad, Hercules, CA, USA) was used to perform the analysis.

### Western blotting

Cells were treated with compound or DMSO, harvested, and lysed in lysis buffer. Proteins from each treatment group were SDS-PAGE separated, transferred to blotting membranes, and blocked with 5% skim milk for one hour. Membranes were incubated with primary antibodies (Supplementary Table S2) for 2 h at room temperature followed by the incubation with horseradish peroxidase-conjugated secondary antibodies (Thermo Fisher Scientific) for 1 h at room temperature. Chemiluminescence imaging was used to find protein bands. Using Multi-Gauge 3.0, the density of the α-tubulin, GAPDH or β-actin bands in each sample was normalized to give the relative density of the bands. Values were displayed using densitometry units that were arbitrarily chosen to represent signal intensity.

### Immunoprecipitation

Immunoprecipitation was performed using lysates from CaCo2/EV cells that were incubated with antibodies overnight at 4 °C and pulled down with Protein A/G Sepharose (Thermoscientific, Rockford, IL, USA) for 3 h. The immunoprecipitated proteins were washed twice with the same buffer, and bound proteins were resolved by SDS-PAGE followed by immunoblotting.

### Bioenergetic analysis by Seahorse instrument

To measure real-time changes in extracellular acidification rate and oxygen consumption rate, an XF96 extracellular flux analyzer (Agilent, Santa Clara, CA, USA) was used. First, CaCo2/EV and CaCo2/KITENIN cells were seeded at 1 × 10^4^ cells/well, incubated overnight in culture medium, and then treated with the non-toxic concentrations of chrysophanol for 48 h. On the examination of day, the plated cells were washed with Agilent Seahorse XF DMEM Medium (pH 7.4) and filled with 180 µL with assay medium enriched with glutamine, glucose and sodium pyruvate. 0.5 µM rotenone (Rot) + antimycin A (AA) and 50 mM 2-deoxy-d-glucose (2-DG) were loaded into the hydrated sensor cartridge for glycolytic rate assay and 1 µM oligomycin, 1 µM carbonyl cyanide 4-(trifluoromethoxy) phenylhydrazone (FCCP), and 0.5 µM Rotenone (Rot) + antimycin A (AA) were loaded for Cell Mito Stress Test. Prior to analysis, the cells were incubated for 1 h at 37 °C in a non-CO2 incubator. The results were analyzed using the Wave software (Agilent).

### Metabolite investigation

Organic acid (OA) standards, internal standards (IS; 3,4-methoxybenzoic acid and ^13^C_2_-succinic acid), and trimethylamine (TEA) were purchased from Sigma-Aldrich (St. Louis, MO, USA). Toluene, diethyl ether (DEE), ethyl acetate (EA), and sodium chloride were purchased from Kanto Chemical (Chuo-ku, Tokyo, Japan). Acetonitrile (ACN), distilled water (DW), and methanol were purchased from J.T. Baker Inc. (Phillipsburg, NJ, USA). Sodium hydroxide (NaOH) and sulfuric acid were purchased from Daejung Reagents Chemicals (Siheung, South Korea). N-Methyl-N-(*tert*-butyldimethylsilyl) trifluoroacetamide (MTBSTFA) + 1% *tert*-butyldimethylchlorosilane and O-Methoxyamine hydrochloride were obtained from Thermo Scientific (Bellefonte, PA, USA). All chemicals were analytical grade.

OA profiling analysis was conducted using GCMS-TQ8040 (Shimadzu Corp., Kyoto, Japan), which was equipped with an Ultra-2 (5% phenyl-95% methylpolysiloxane bonded phase; 25 m × 0.20 mm i.d., 0.11 um film thickness) cross-linked capillary column (Agilent Technologies, USA) and interfaced with a triple quadrupole mass spectrometer. The samples were introduced in a split-injection mode (10:1). The interface, injector, and ion source were adjusted at 300, 260, and 230 ℃, respectively. In a linear velocity flow control mode, helium was used as the carrier gas at a flow rate of 0.5 mL/min. The collision gas used was argon. The oven temperature was set as follows: 100 ℃ for 2 min, 100–300 ℃ at 10 ℃/min, and then held for 8 min. GC–MS/MS was utilized in the multiple reaction monitoring mode. The collision energy differed from 5 to 50 V in increments of 5 V.

Cell and media samples were subjected to methoxime/*tert*-butyldimethylsilyl (MO/TBDMS) derivatives for OA profiling analysis, as described in our previous studies [13–17]. In brief, ACN was added to the lysed cell (2.4 × 10^6^) and media (30 μL) samples, including ISs (^13^C^2^-succinic acid and 3,4-methoxybenzoic acid), for deproteinization. The mixture was then centrifuged at 12,300 × g for 3 min. Afterward, it was transferred to DW, and the aqueous phase was alkalized to pH ≥ 12 with 5 M NaOH. The mixture was subjected to the methoximation with *O*-methoxyamine hydrochloride (1 mg) at 60 ℃ for 60 min. The aqueous phase was acidified to pH ≤ 2 using 10% sulfuric acid and saturated with sodium chloride. Liquid–liquid extraction was carried out using 3 mL of DEE and 2 mL of EA, respectively. After adding 5 μL of TEA, the extracts were evaporated to dryness under a gentle stream of nitrogen at 40 ℃. The extracts were reacted with toluene (10 μL) and MTBSTFA (20 μL) for 60 min at 60 ℃ for TBDMS derivative formation. Then, 1 μL was introduced for GC–MS/MS analysis.

The concentrations of 15 OAs were determined using their respective standard calibration curves. The mean concentrations of each OA in the cell and media samples were normalized to the corresponding mean values in the control groups [18–22].

### Docking study

3-dimensional structures of ErbB4 kinase are available in RCSB database (http://rcsb.org); type I (PDB ID: 3BBT) as inactive state and type II (PDB ID: 3BCE) as active state. The missing loops (active loop 844–857) of X-ray were modeled by alphafold. The ligands were built by Marvinsketch. Explicit hydrogens and atomic charges of target protein and ligands were added by Chimera software. The ATP binding site was assigned by 25 Å cubic box from center of mass of the lapatinib bound to the ErbB4 kinase. All docking simulations were performed by Vina 1.1.2 [23]. The docking structures were analyzed by LigPlot + and represented by Chimera software [24, 25].

### Statistical analysis

Data represents as means ± standard deviation. All statistical analyses were carried out using the Sigma Plot software.

For metabolite analysis in supplementary Fig. 1 and 2, concentration data from cell and media samples showed as the mean ± standard deviation (SD). After transforming the concentration data to log10-transformed data, Wilcoxon rank-sum test, a non-parametric statistical test, was conducted to compare the distribution of two independent groups. Statistically significant results are shown by P-value < 0.05. Multivariate statistical analyses of metabolomic data, including principal component analysis (PCA), orthogonal partial least squares discriminant analysis (OPLS-DA), and hierarchical clustering heatmap analysis as cluster analysis, were performed using MetaboAnalyst 5.0 (https://www.metaboanalyst.ca) [26–29].

## Results

### Chrysophanol reduces KITENIN-mediated cell motility

**Fig. 1:**
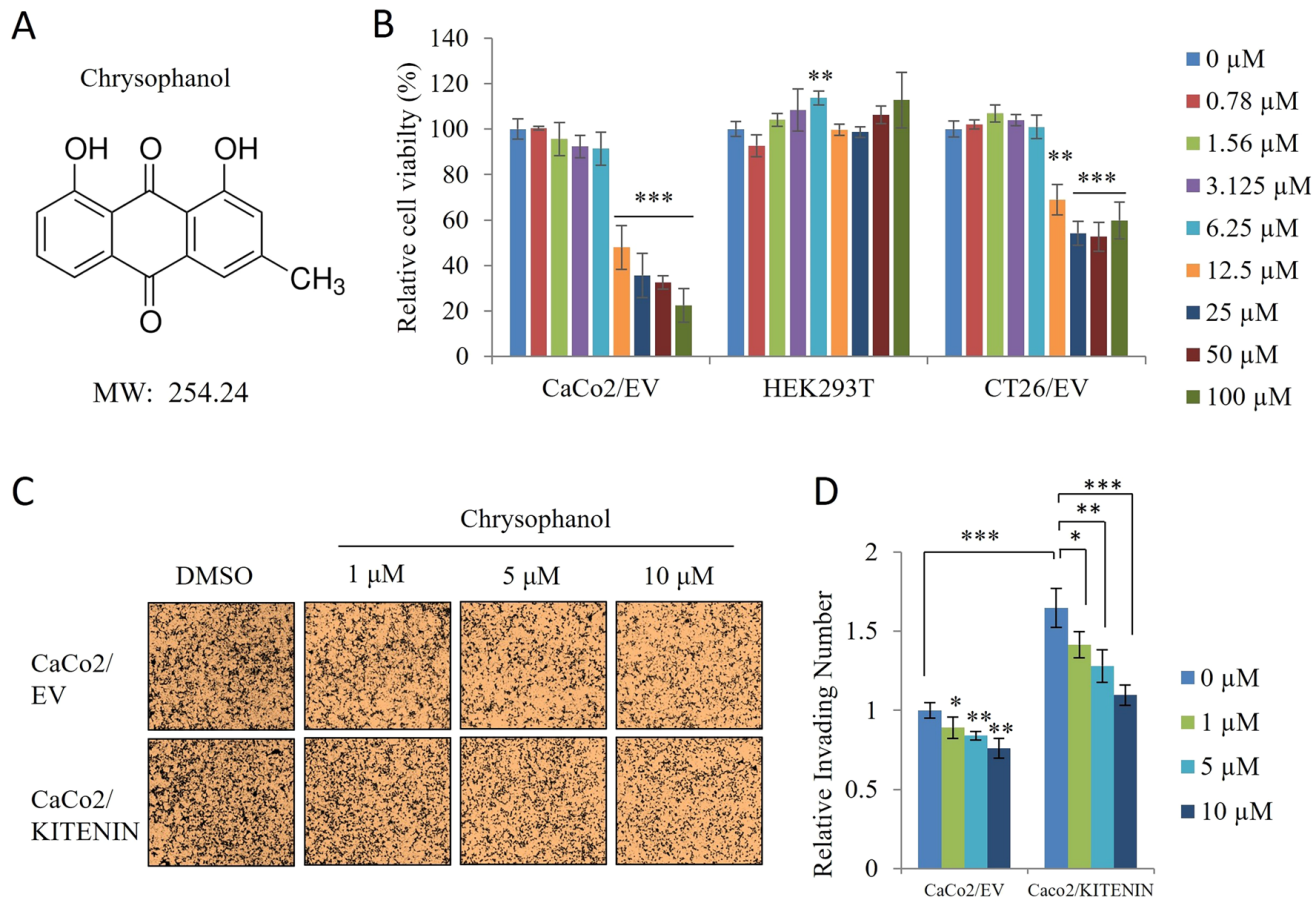
Chrysophanol inhibits KITENIN-mediated cell invasion. **A** Structure of the chrysophanol. **B** The effect of chrysophanol on the viability of CaCo2/empty vector (EV), HEK293T and CT26/EV cells. Cells were seeded in 96-well plates. After incubation for 12 h, the cells were treated with the indicated concentrations of chrysophanol for 48 h. Cell viabilities were measured by an MTT assay. **C**, **D** Chrysophanol inhibits the upregulated cell invasion mediated by KITENIN overexpression. CaCo2 cells were transfected with empty vector or KITENIN-V5 and treated with DMSO or chrysophanol (1, 5, and 10 μM) and subjected to an in vitro Transwell invasion assay. The histogram represents the relative number of invading cells. Data represent means ± standard deviation; * p < 0.05; ** p < 0.01; *** p < 0.001

Chrysophanol was tested in a cell viability assay to identify non-cytotoxic concentrations (Fig. 1A, B). Based on the results, we used concentrations of 1, 5, and 10 µM in subsequent experiments. A study that used clinical data reported that KITENIN expression was significantly higher in the colon cancer tissues of patients with stage 3 and 4 disease than in tissue from patients with stage 1 disease and corresponding metastatic tissues [30]. Based on this finding, we used CaCo2/empty vector and CaCo2/KITENIN overexpression cell lines to test the effects of chrysophanol on cell motility. Our results showed that chrysophanol inhibited KITENIN-mediated cell motility in a concentration-dependent manner (Fig. 1C, D).

### Chrysophanol targets the KITENIN/ErbB4 complex

**Fig. 2:**
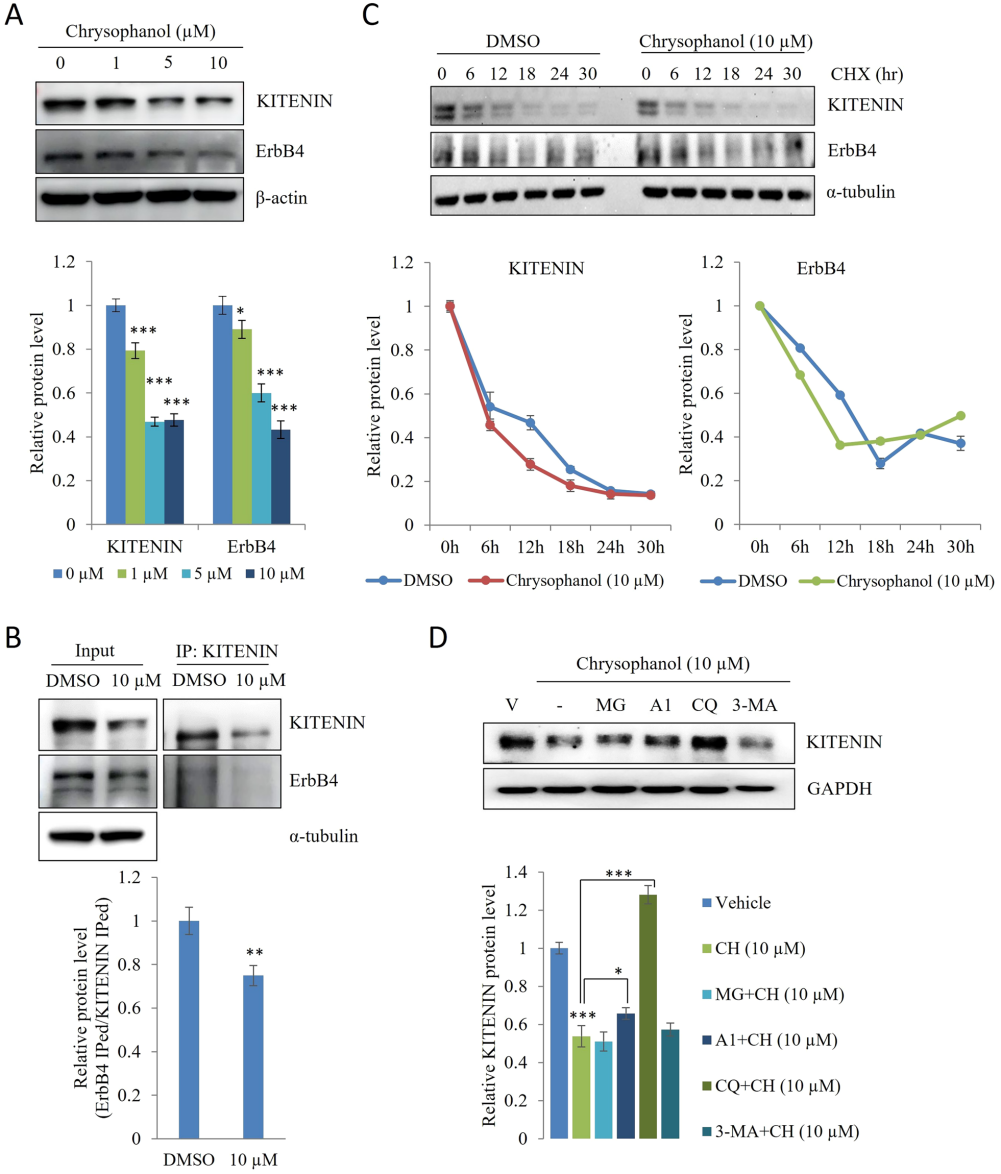
Chrysophanol downregulates KITENIN and ErbB4 levels and induces autophagic degradation of KITENIN. **A** Protein levels of KITENIN and ErbB4 in CaCo2/EV cells treated with 1, 5, and 10 μM chrysophanol for 48 h. **B** To test whether the treatment reduces the interaction between KITENIN and ErbB4, samples were subjected to immunoprecipitation using anti-KITENIN antibody. Data show the relative ErbB4 Iped/KITENIN Iped ratio relative to DMSO for the DMSO and treatment groups. **C** CaCo2/EV cells treated with DMSO or chrysophanol (10 μM). The protein levels of ErbB4 and KITENIN were determined after treatment with cycloheximide at the indicated times. The quantitative analysis of protein expression is given. **D** CaCo2/EV cells were pretreated with vehicle, the proteasome inhibitor MG132 (MG, 10 μM), the lysosomal degradation inhibitors bafilomycin A1 (A1, 100 nM) and chloroquine (CQ, 10 μM), or the autophagosome blocker type III phosphatidylinositol 3-kinase inhibitor (3-MA, 10 μM), and later treated with a high concentration of chrysophanol (10 μM). Levels of KITENIN were examined by immunoblot analyses. β-actin, GAPDH or α-tubulin served as a loading control. Data represent means ± standard deviation; * p < 0.05; ** p < 0.01; *** p < 0.001

Next, we examined protein levels of KITENIN and ErbB4 and showed that chrysophanol suppressed KITENIN and ErbB4 protein levels (Fig. 2A). Our previous study identified that KITENIN and ErbB4 are co-expressed in advanced human CRC tissues, KITENIN stabilizes phospho-ErbB4 levels, and ErbB4 is an unconventional binding partner of KITENIN in the plasma membrane [7]. Based on this information, we performed an immunoprecipitation assay using a KITENIN antibody on cell lysates treated with chrysophanol or DMSO to determine whether the treatment disrupts the interaction between ErbB4 and KITENIN. The results showed that chrysophanol reduced the interaction between KITENIN and ErbB4 (Fig. 2B). Then, we used the protein synthesis inhibitor cycloheximide to evaluate the protein stability of KITENIN and ErbB4 following chrysophanol treatment. The results showed that the stability of KITENIN and ErbB4 proteins was impaired earlier in the treatment group than in the control group (Fig. 2C). However, surprisingly, ErbB4 protein level returned after 24 h of cycloheximide treatment in pre-treated chrysophanol condition. In this case, compensatory mechanisms, feedback mechanisms to maintain homeostasis, and post-translational modifications may have been effective in the ErbB4 protein stabilization experiment. These factors suggest that ErbB4 may have reversed protein degradation as an initial disruption, causing it to return after 24 h, although KITENIN levels continued to decline after 24 h. To determine whether autophagy induction or other proteolytic pathways are required for the destruction of KITENIN by chrysophanol, we performed an assay that subjected cells to proteasomal or lysosomal degradation inhibitors. As shown in Fig. 2D, chloroquine (10 µM) prevented the degradation of KITENIN by a high concentration of chrysophanol (10 µM). This observation suggests that chrysophanol degrades KITENIN via an autophagy pathway.

**Fig. 3:**
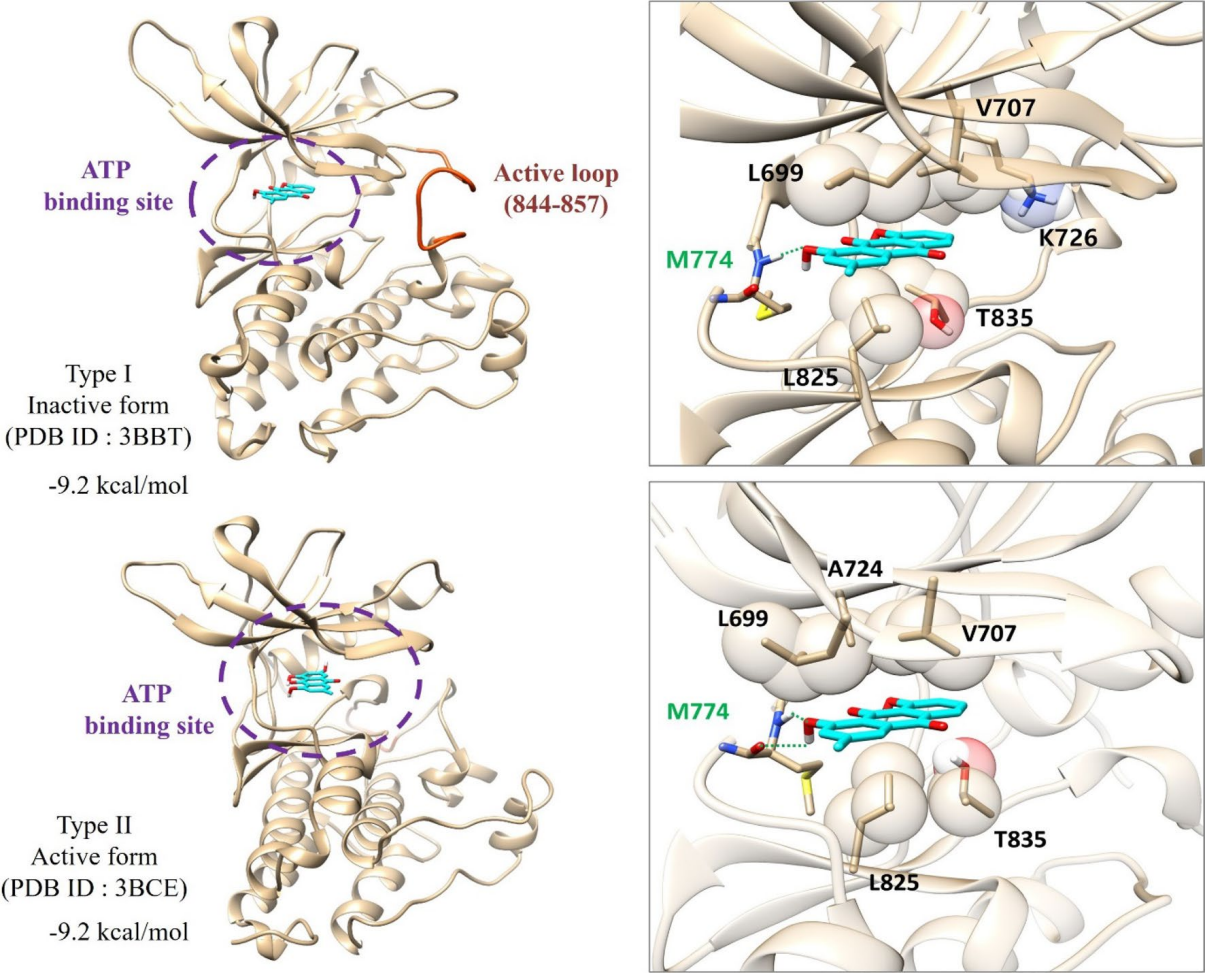
Chrysophanol binds to ErbB4. The predicted interactions of chrysophanol with the inactive (Type I, PDB ID: 3BBT) and active (Type II, PDB ID: 3BCE) form of ErbB4 using Vina 1.1.2

To further characterize the interaction between chrysophanol and ErbB4, we calculated binding affinities using a docking simulation. The inactive form (type I, PDB: 3BBT) and active form (type II, PDB: 3BCE) of ErbB4 were used, and binding affinities of chrysophanol for both forms were calculated as − 9.2 kcal/mol. The results showed that chrysophanol binds to ErbB4 via residues M774, L699, A724, V707, T835, L825, and K726 (Fig. 3). Chrysophanol makes a hydrogen bond to M774 of the hinge lobe and is located in the ATP-binding site regardless of the diverse conformational change such as inactive state and active state of the active loop (residue 844–857).

### Chrysophanol inhibits aerobic glycolysis in CRC by targeting the KITENIN/ErbB4 complex

**Fig. 4:**
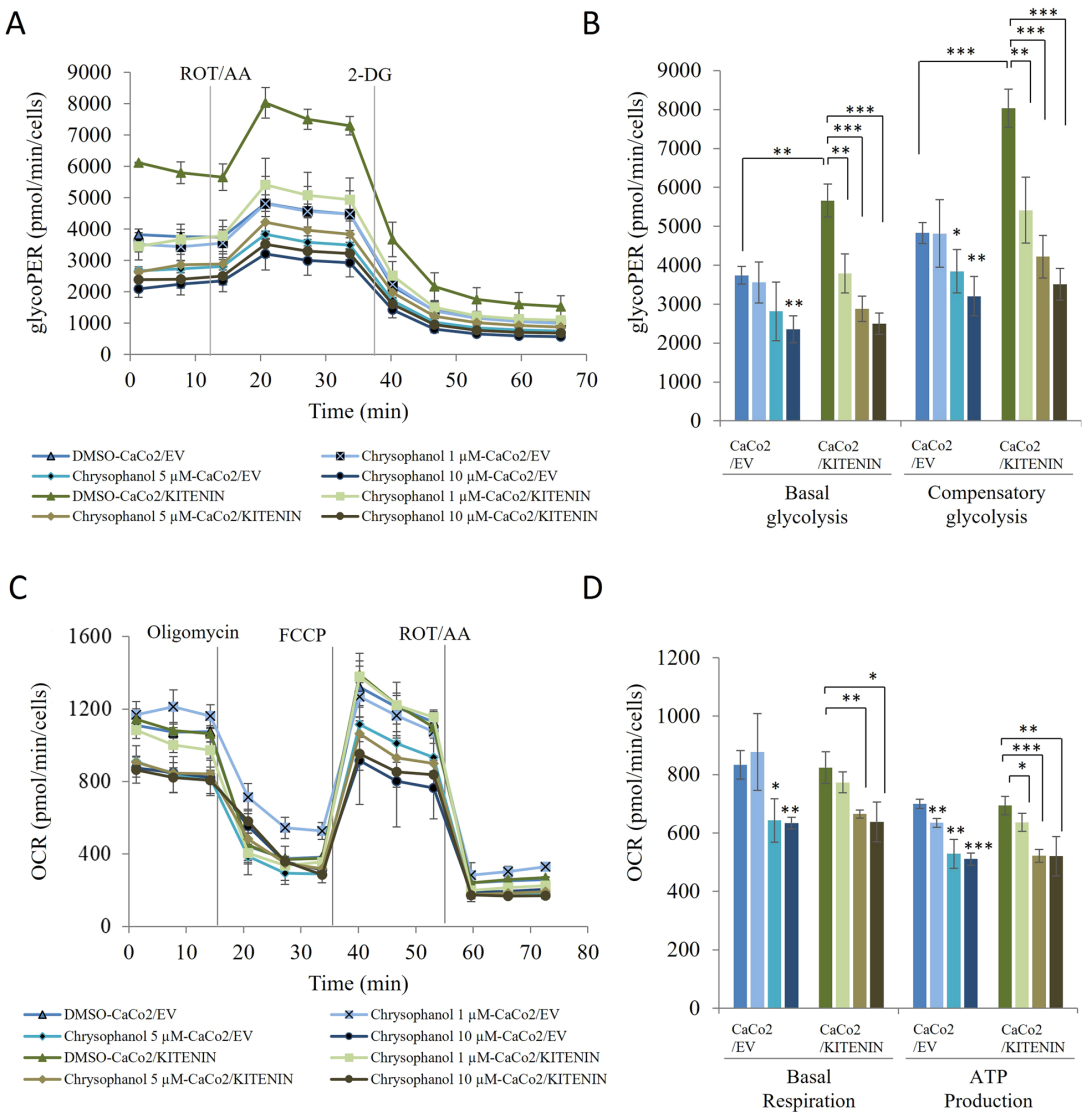
Chrysophanol downregulates KITENIN-induced glycoPER, and oxygen consumption rate. CaCo2/EV and CaCo2/KITENIN cells were treated with chrysophanol for 48 h and then analyzed using a Seahorse glycolytic rate assay kit or a Seahorse cell mito stress kit. **A**, **B** glycoPER, basal glycolysis, and compensatory glycolysis levels. **C**, **D** Oxygen consumption rate, basal respiration, and ATP production levels. Data represent means ± standard deviation; * p < 0.05; ** p < 0.01; *** p < 0.001

In our previous study, we showed that KITENIN upregulates the expression of genes associated with aerobic glycolysis and the glycolytic proton efflux rate (glycoPER) [31]. Here, we tested KITENIN-mediated aerobic glycolysis using the Seahorse XF glycolytic rate assay, which measures cellular glycoPER using both the extracellular acidification rate and the oxygen consumption rate. In this assay, antimycin/rotenone mix (ROT/AA), a mitochondrial electron transport chain inhibitor, is first used to inhibit mitochondrial oxygen consumption. Then, glucose analog 2-deoxy-D-glucose (2-DG) is used to slow glycolysis by competing with the first enzyme in the glycolytic pathway, glucose hexokinase. The resultant decrease in the proton efflux rate gives a qualitative indication that the proton efflux rate produced prior to ROT/AA addition is primarily attributable to glycolysis [32, 33]. Our results showed that, chrysophanol treatment downregulates KITENIN-induced values of basal glycolysis and compensatory glycolysis after a glycolysis rate assay in CRC cells. To further characterize the effect of chrysophanol on energy metabolism, we used the Seahorse cell mito stress test kit in CaCo2/empty vector and CaCo2/KITENIN cells. The first compound injected in this assay is oligomycin, which rapidly hyperpolarizes the mitochondrial membrane by inhibiting ATP synthesis, thus preventing the passage of protons from mitochondrial complexes. By transferring protons across the mitochondrial inner membrane, the second injection of carbonyl cyanide-4 (trifluoromethoxy)phenylhydrazone (FCCP) restores the hyperpolarization generated by oligomycin. Finally, ROT/AA injection completely stops mitochondrial respiration by inhibiting mitochondrial complexes I and III [34]. The results of this assay can determine parameters such as basal respiration and ATP production. We found that chrysophanol downregulated basal respiration and ATP production in CaCo2/empty vector and CaCo2/KITENIN cells. From our data, we conclude that chrysophanol has potential therapeutic effects on cancer energy metabolism, including KITENIN-mediated aerobic glycolysis (Fig. 4).

### Chrysophanol suppresses the levels of key markers related to KITENIN-induced aerobic glycolysis

**Fig. 5:**
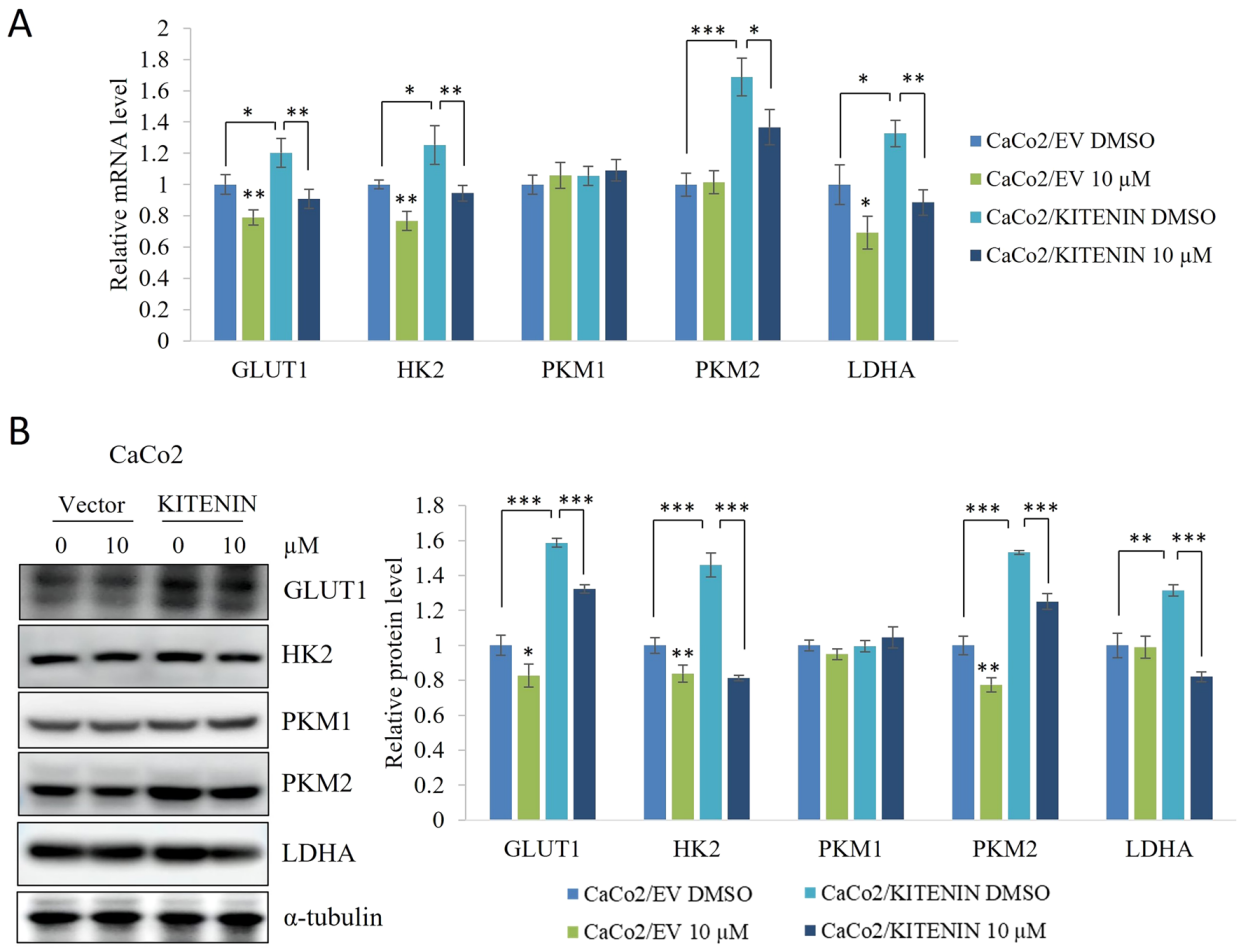
KITENIN-mediated increases in aerobic glycolysis markers are reduced by chrysophanol. CaCo2/EV and CaCo2/KITENIN cells were treated with chrysophanol for 48 h and then used for qRT-PCR or Western blotting. The mRNA expression (**A**) and protein levels (**B**) of Glut1, HK2, PKM1, PKM2, and LDHA were determined. The relative protein levels are shown. α-tubulin served as a loading control. Data represent means ± standard deviation; * p < 0.05; ** p < 0.01; *** p < 0.001

In CRC, glucose absorption and aerobic glycolysis rates are typically elevated because they benefit tumor formation. The high glycolysis rate in tumor cells is dependent on essential metabolic enzymes and substrates, which are processed through complex regulatory networks. Glycolysis is the process by which glucose is converted to pyruvate, ATP, and NADH (nicotinamide adenine dinucleotide) in cells [35, 36]. Next, we determined the effects of chrysophanol on KITENIN-mediated GLUT1, HK2, PKM1, PKM2, and LDHA mRNA and protein levels. Our results showed that chrysophanol had a greater effect on the expression of most of these aerobic glycolysis markers in CaCo2/KITENIN cells than in CaCo2/empty vector cells (Fig. 5). However, chrysophanol did not change the levels of PKM1 in either cell type. These findings provide further evidence that chrysophanol attenuates KITENIN/ErbB4-mediated signaling.

### Chrysophanol downregulates important metabolites in the KITENIN overexpression cell model

**Fig. 6:**
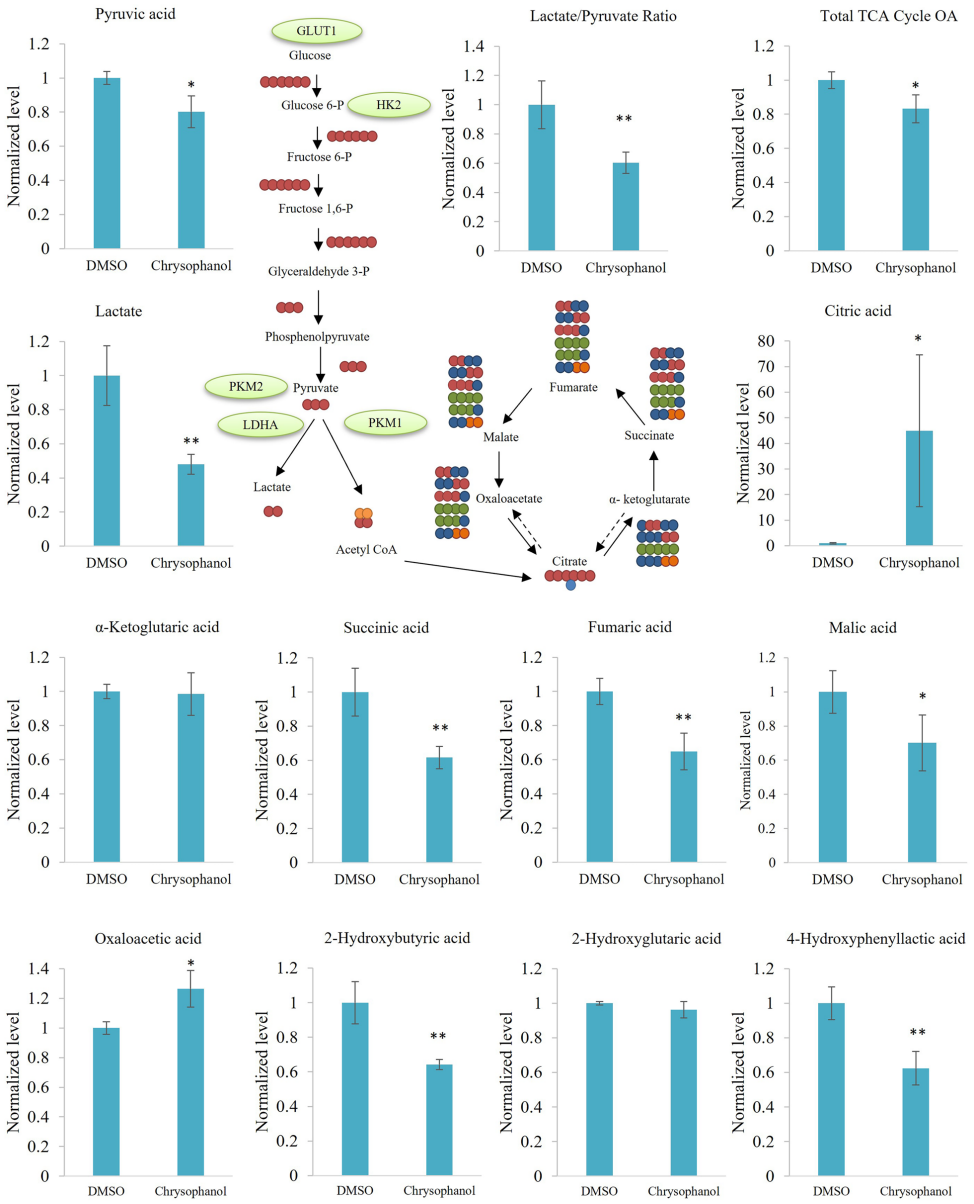
The production of metabolites of aerobic glycolysis and oxidative phosphorylation is suppressed by chrysophanol. CaCo2/KITENIN cells were treated with chrysophanol 10 μM for 48 h. Then, control and treatment group samples were collected and analyzed by GC–MS to determine levels of pyruvic acid, lactate, lactate/pyruvic acid, total TCA cycle organic acids (OAs), citric acid, α-Ketoglutaric acid, succinic acid, fumaric acid, malic acid, oxaloacetic acid, 2-hydroxybutyric acid, 2-hydroxyglutaric acid, and 4-hydroxyphenyllactic acid. Data represent means ± standard deviation. * p < 0.05; ** p < 0.01; *** p < 0.001

PKM2 has a greater affinity for its substrate phosphoenolpyruvate and a higher pyruvate kinase enzymatic activity when it is in a tetrameric configuration, which allows it to catalyze the generation of pyruvate from phosphoenolpyruvate. In the absence of environmental stress, PKM2 primarily exists as a dimer and has a modest pyruvate kinase activity [37, 38]. The excessive use of glucose by tumor cells is followed by the uncontrolled generation of lactic acid and hydrogen ions (H +), which lowers the pH of the surrounding environment. The most prevalent metabolite in the microenvironment of highly glycolytic tumors is lactate [39, 40]. Our results showed that chrysophanol suppressed the levels of pyruvate and lactate and reduced the lactate/pyruvic acid ratio in CaCo2/KITENIN cells. In addition, chrysophanol inhibited total TCA cycle organic acids. However, it is known that there is a buildup of metabolic by-products in tumor cells, which suggests that the TCA cycle was not completely inhibited but rather was disrupted. It is reported that the TCA cycle is interrupted, with the first major point being from citrate to α-ketoglutarate and the second major point being from succinate to fumarate [41–43]. We found that chrysophanol increased levels of the citric acid and oxaloacetic acid but reduced levels of other metabolites, namely succinic acid, fumaric acid, and malic acid. There was no change in α-ketoglutaric acid levels. In addition, while the levels of 2-hydroxybutyric acid and 4-hydroxyphenyllactic acid metabolites from other oncometabolites were downregulated by chrysophanol, no significant change was observed in 2-hydroxyglutaric acid levels (Fig. 6).

### Chrysophanol downregulates transcriptional regulators of energy metabolism in the KITENIN overexpression cell model

**Fig. 7:**
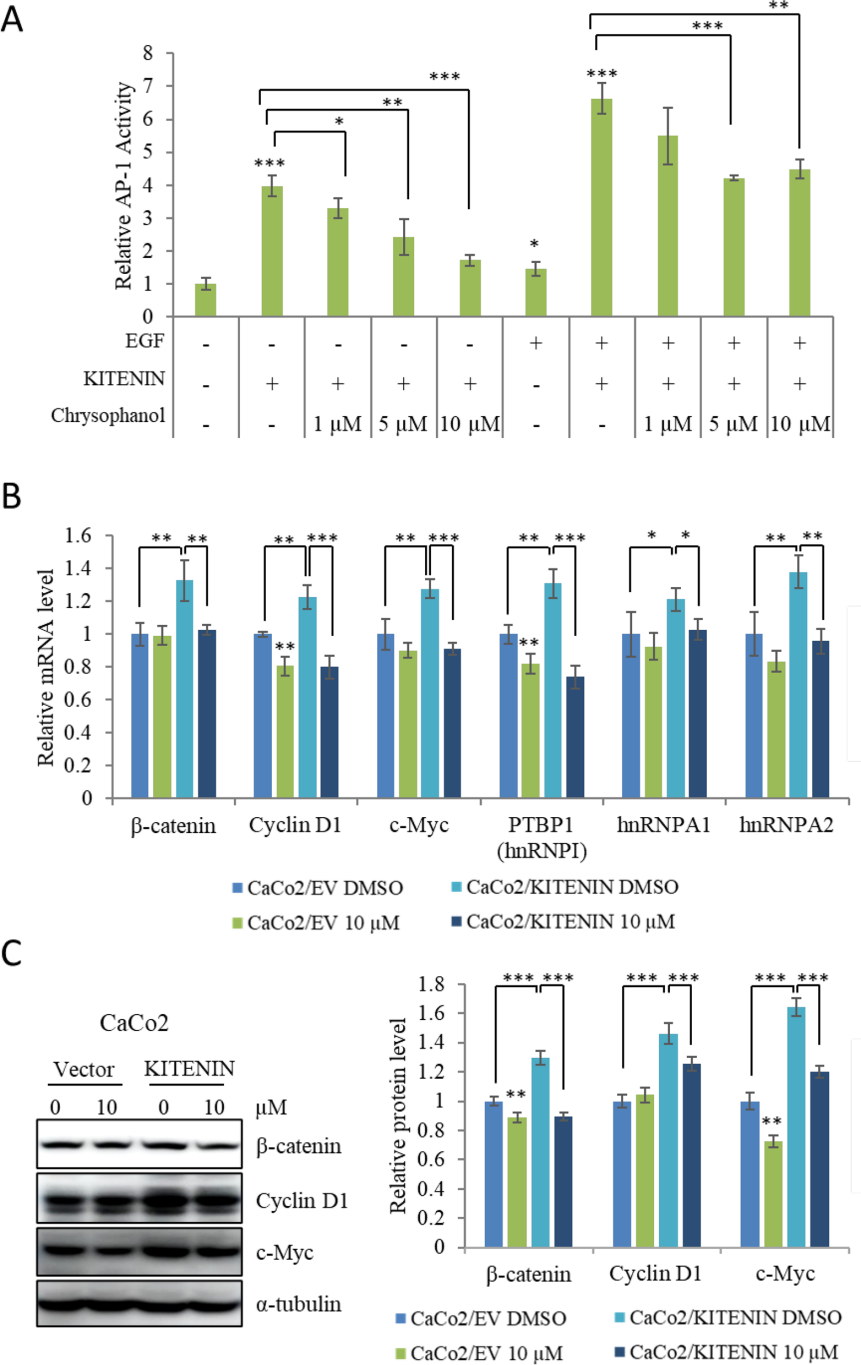
Downstream effectors of the KITENIN/ErbB4 complex are downregulated by chrysophanol. **A** Chrysophanol reduced the promoter activity of the AP-1 that is regulated by KITENIN. HEK293T cells were treated with different concentrations of chrysophanol with or without EGF for 48 h after 12 h of transfection with KITENIN and the AP-1 reporter plasmid. **B** Quantitative analysis of the mRNA expression of β-catenin, cyclin D1, c-Myc, hnRNPI, hnRNPA1, and hnRNPA2 in CaCo2/EV and CaCo2/KITENIN cells treated with 10 µM chrysophanol for 48 h. **C** Western-blot analysis of β-catenin, cyclin D1, and c-Myc in CaCo2/EV and CaCo2/KITENIN cells treated with 10 µM chrysophanol for 48 h. The relative levels of the indicated protein are shown in the bar graphs. Data represent means ± standard deviation. * p < 0.05; ** p < 0.01; *** p < 0.001

Signaling downstream of the KITENIN/ErbB4 complex upregulates the activating protein-1 (AP-1) and T-cell factor (TCF) axis, and increases expression of β-catenin, cyclin D1, c-Myc, hnRNPI, hnRNPA1, and hnRNPA2, which are associated with metabolic activity [31]. First, we determined KITENIN-mediated AP-1 activity in the presence and absence of EGF in HEK293T cells. Our results showed that chrysophanol had a concentration-dependent inhibitory effect (Fig. 7A). Further investigation tested the effects of chrysophanol on transcriptional regulators related to the AP-1 axis. Our results showed that chrysophanol significantly downregulated the mRNA levels of β-catenin, cyclin D1, c-Myc, hnRNPI, hnRNPA1, and hnRNPA2 in Caco2/KITENIN cells (Fig. 7B). Chrysophanol also inhibited protein levels of β-catenin, c-Myc, and cyclin D1 to a greater level in KITENIN overexpressing cells than in control cells (Fig. 7C). Together, these results show that chrysophanol inhibits transcriptional factors that are regulated by KITENIN.

## Discussion

Genetic background, lifestyle, and environmental risk factors contribute to the risk of CRC. There are more than 150,000 new cases of CRC and more than 50,000 deaths each year worldwide [44, 45]. Genetic mutations such as KRAS, APC, TP53, SMAD4, EGFR, and MYC regulate cancer processes in CRC by influencing cancer cell motility and metabolic reprogramming, including aerobic glycolysis [46–54]. In addition, oncogenic receptor tyrosine kinase signaling participates in cancer metabolism, promoting cell growth and production of cellular metabolites [55]. Targeted therapies are used in many types of cancers, including CRC, that originate from abnormal tyrosine kinase activity [56]. Cetuximab and panitumumab are EGF receptor-targeted drugs, while encorafenib and regorafenib target signaling downstream of EGF receptor signaling. Yet, as we have shown in our previous study, cetuximab has a poor response on KITENIN/ErbB4 signaling in CRC cells [9]. Although these treatments have been clinically used and improved patient outcomes in recent years, the development of drug resistance necessitates the need for new and alternative treatments for CRC [57].

The current study shows that in a model of CRC, the effects of the KITENIN/ErbB4 oncogenic complex, which in previous reports were shown to modulate cell motility and aerobic glycolysis, are suppressed by chrysophanol, a compound that cleaves the KITENIN/ErbB4 complex. The findings showed that chrysophanol (1) suppresses KITENIN-mediated cell invasion; (2) destabilizes KITENIN and downregulates protein levels of ErbB4 and KITENIN via autophagic degradation; (3) binds to ErbB4 and blocks the interaction between KITENIN and ErbB4; (4) suppresses KITENIN-mediated changes in glycolysis, levels of pyruvate and lactate acid and the lactate/pyruvic acid ratio; (5) suppresses mitochondrial respiration and levels of TCA cycle metabolites; and (6) suppresses the expression levels of KITENIN-mediated downstream effectors. Collectively, these results demonstrate that the oncogenic activity of the KITENIN/ErbB4 complex is suppressed by chrysophanol, preventing the progression of CRC.

The expression of KITENIN plays an important role in the progression of CRC at all stages and is associated with lymph-node metastasis. KITENIN expression is associated with MAPK signaling and the AP-1 axis, and knockdown of KITENIN downregulates markers of cyclin D1, COX2, MMP3 and ERK1/2, HIF-1A, EMT, stemness and aerobic glycolysis, suppressing cancer progression [31, 58–60]. The correlation between KITENIN expression, which is an oncogenic biomarker, and WNT signaling in CRC has been known for many years, and a recent report shows that this correlation also occurs in glioblastoma [6]. The KITENIN/ErbB4–Dvl2 interaction is well known, and in recent years, new binding partners of KITENIN oncogenic function have been elucidated [7, 8, 30]. KITENIN interacts with myosin-X (Myo10), which functions to stabilize the KITENIN homodimer [3]. Our recent study described the KITENIN–KSRP interaction and showed that RACK1 is also a part of this complex. KITENIN forms a functional complex with several partners, including ErbB4 [61]. The oncogenic action of the KITENIN/ErbB4 complex is associated with the expression of MYO1D. MYO1D binds to the kinase domain of the EGF receptor family (except ErbB3), causing KITENIN and ErbB4 to attach to the plasma membrane. These reasons make the KITENIN/ErbB4 complex a potential therapeutic target. There have been efforts to suppress the oncogenic activity of the KITENIN complex. DKC1125 and DKC-C14S, which are KSRP binding molecules, disintegrate the KITENIN complex and act on KITENIN-mediated metastasis, aerobic glycolysis, and tumorigenesis [31, 61]. Lichen secondary metabolites (usnic, atranorin, and physciosporin) and a marine-derived metabolite (marinobazzanan) suppress KITENIN expression levels [62–65]. A KITENIN dimerization-interfering peptide suppresses CRC metastasis and progression [3]. There are several ErbB4 inhibitors, including allitinib, poziotinib, dacomitinib, lapatinib, afatinib, canertinib, neratinib, pyrotinib, and ibrutinib. The majority of these inhibitors bind to the ATP-binding site covalently and irreversibly. While this property increases their potency, it also increases the risk of severe side effects [66, 67].

Chrysophanol has antidiabetic, anticancer, neuroprotective, hepatoprotective, anti-ulcer, anti-inflammatory, anti-viral, and anti-fungal effects, as well as other miscellaneous activities [68]. Studies on the anticancer activity of chrysophanol have generally focused on apoptotic cell death and the regulation of caspases 3 and 9, PARP, Bax, and Bcl2 levels [10, 69–71]. Along with these studies, other studies have shown that chrysophanol regulates PI3K/AKT, MAPK, ERK1/2, JNK, and c-Jun in cellular signaling [70–73]. The current study focused on the effects of chrysophanol on cell motility and cancer metabolism in CRC via the KITENIN and ErbB4 pathways, and the results provide a new perspective on the previously demonstrated anticancer effects of chrysophanol.

Glucose transporters facilitate glucose uptake in CRC [74]. The initial stage of glucose metabolism, the phosphorylation of glucose to glucose 6-phosphate (G6P), is catalyzed by hexokinases. Increasing evidence suggests that HK2 is critical for cell survival in addition to its key role in glycolysis [75]. Pyruvate kinase M (PKM) controls the rate-limiting phase in glycolysis and upholds a sophisticated regulatory network. PKM2 has a greater contribution to the development of CRC than PKM1; both these molecules are splice variants encoded by the same PK gene [76]. Due to the high levels of lactate present it, LDHA (lactate dehydrogenase A) is intimately associated with the emergence of cancer. The progression of CRC is determined by the sustained self-renewal of stem cells [77]. In a previous study, we found that KITENIN selectively upregulates PKM2 expression via c-Myc, by upregulating hnRNPI, hnRNPA1 and hnRNPA2 expression, and increasing nuclear PKM2 levels, causing PKM2 to act as a co-transcriptional factor [31]. The current results show that transcription factors and glycolytic enzymes upregulated by KITENIN are downregulated by chrysophanol, providing further evidence that chrysophanol targets the KITENIN complex.

A previous study showed that KITENIN serves as a scaffolding molecule that simultaneously recruits protein kinase C delta (PKC delta) and Disheveled (Dvl) to its membrane-spanning C-terminal region. This scaffolding function enables KITENIN to form a complex that activates ERK/AP-1 via a PKC delta component and organizes actin filaments via a Dvl component [78]. The amount of GLUT1 that is stored in the membrane is regulated by PKC-mediated GLUT1 S226 phosphorylation, which controls the speed of glucose transport [79]. In addition, under conditions of high KITENIN expression, the ErbB4 CYT-2 isoform has the largest impact on the activation of AP-1 by EGF [7]. AP-1 target genes transcribe genes associated with metabolism. Metastatic malignancies have higher glycolytic fluxes because c-Myc upregulates several glycolytic genes (HKII, PFK-1, TPI, GAPDH, ENO, LDHA, and MCT1) at the metabolic level [53, 80–82]. A previous study investigated whether increased expression of the KITENIN/ErbB4 complex promotes intestinal adenoma in the tumor microenvironment associated with APC loss. The study generated villin–KITENIN transgenic mice, and immunohistochemical analyzes demonstrated ErbB4, c-Jun and nuclear β-catenin levels were increased in these mice [7]. There are reports showing that PKM2 is involved in the transactivation of β-catenin. Moreover, the nuclear translocation of PKM2 causes PKM2 to bind the c-Src phosphorylated Y333 residue of β-catenin [83, 84]. The resulting protein complex controls the production of cyclin D1 by attaching to the cyclin D1 promoter region [83, 85]. In addition, the expressions of markers, including cyclin D1, which is an AP-1 target gene, are significantly upregulated in human CRC tissues compared with normal mucosa tissues [86]. The current results show that downstream regulators of KITENIN and the AP-1 and TCF4 target genes involved in metabolic signaling are downregulated by chrysophanol.

## Conclusions

In conclusion, we report that KITENIN expression and its functional oncogenic complex involved in cancer progression were suppressed by chrysophanol. Chrysophanol treatment may be a strategy to overcome the oncogenic functions of KITENIN, which is expressed in many types of cancer, and is associated with poor prognosis, metastasis, tumorigenesis, and aerobic glycolysis.

### Supplementary Information

Supplementary Material 1: Supplementary Figure 1. PCA, OPLS-DA, VIP score, and heatmap analysis for CaCo2/KITENIN cell with DMSO and chrysophanol treatment. Supplementary Figure 2. PCA, OPLS-DA, VIP score, and heatmap analysis for CaCo2/KITENIN media samples treatment by DMSO and chrysophanol treatment. Supplementary Table 1. qRT-PCR primers. Supplementary Table 2. Antibody information.

## Data Availability

The data and materials of the study can be obtained from the corresponding author upon request.

## Abbreviations

AP-1: Activator protein 1
CRC: Colorectal cancer
ECAR: Extracellular acidification rate
EGF: Epidermal growth factor
ERK: Extracellular signal-regulated kinase
glycoPER: Glycolytic proton flux rate
GLUT1: Glucose transporter 1
HK2: Hexokinase 2
HnRNP: Heterogeneous RNP
OCR: Oxygen consumption rate
KITENIN: KAI1 COOH-terminal interacting tetraspanin
LDHA: Lactate dehydrogenase A
MAPK: Mitogen-activated protein kinase
PEP: Phosphoenolpyruvate
PKC: Protein kinase C
PKM: Pyruvate kinase M
TCA: Tricarboxylic cycle
VANGL: Van Gogh-like protein
