# Supplementary material for: Chrysophanol inhibits of colorectal cancer cell motility and energy metabolism by targeting the KITENIN/ErbB4 oncogenic complex
Source: Cancer Cell Int. 2024 Jul 20;24:253. doi: 10.1186/s12935-024-03434-x (PMC11264950; doi:10.1186/s12935-024-03434-x)
Supplement: Supplementary file 1 — Supplementary Material 1: Supplementary Figure 1. PCA, OPLS-DA, VIP score, and heatmap analysis for CaCo2/KITENIN cell with DMSO and chrysophanol treatment. Supplementary Figure 2. PCA, OPLS-DA, VIP score, and heatmap analysis for CaCo2/KITENIN media samples treatment by DMSO and chrysophanol treatment. Supplementary Table 1. qRT-PCR primers. Supplementary Table 2. Antibody information. [file 12935_2024_3434_MOESM1_ESM.docx]

Supplementary Information

**Chrysophanol inhibits of colorectal cancer cell motility and energy metabolism by targeting the KITENIN/ErbB4 oncogenic complex**

Mücahit Varlı ^1^, Eunae Kim ^2^, Songjin Oh ^1^, Sultan Pulat ^1^, Rui Zhou ^1^, Chathurika D.B. Gamage ^1^, Barış Gökalsın ^3^, Nüzhet Cenk Sesal ^3^, Kyung Keun Kim ^4^, Man-Jeong Paik ^1^, Hangun Kim ^1^, *

^1^College of Pharmacy, Sunchon National University, Sunchon, Republic of Korea; mucahitvarli@s.scnu.ac.kr (M.V.), osj7797@naver.com (S.O.); sultanpulat@s.scnu.ac.kr (S.P); zhourui274@gmail.com (R.Z.); chathurika.gamage@gmail.com (C.D.B.G.); paik815@sunchon.ac.kr (M.-J.P.).

^2^College of Pharmacy, Chosun University, 146 Chosundae-gil, Gwangju 61452, Republic of Korea; eunaekim@chosun.ac.kr (E.K.).

^3^ Faculty of Arts and Sciences, Department of Biology, Marmara University, Istanbul, Türkiye; baris.gokalsin@marmara.edu.tr (B.G.); cses-al@marmara.edu.tr (N.C.S.).

4 Department of Pharmacology, Chonnam National University Medical School, 160 Baekseoro, Gwangju 61469, Republic of Korea; kimkk@chonnam.ac.kr (K.K.K.).

* Correspondence: hangunkim@scnu.ac.kr (H.K.)


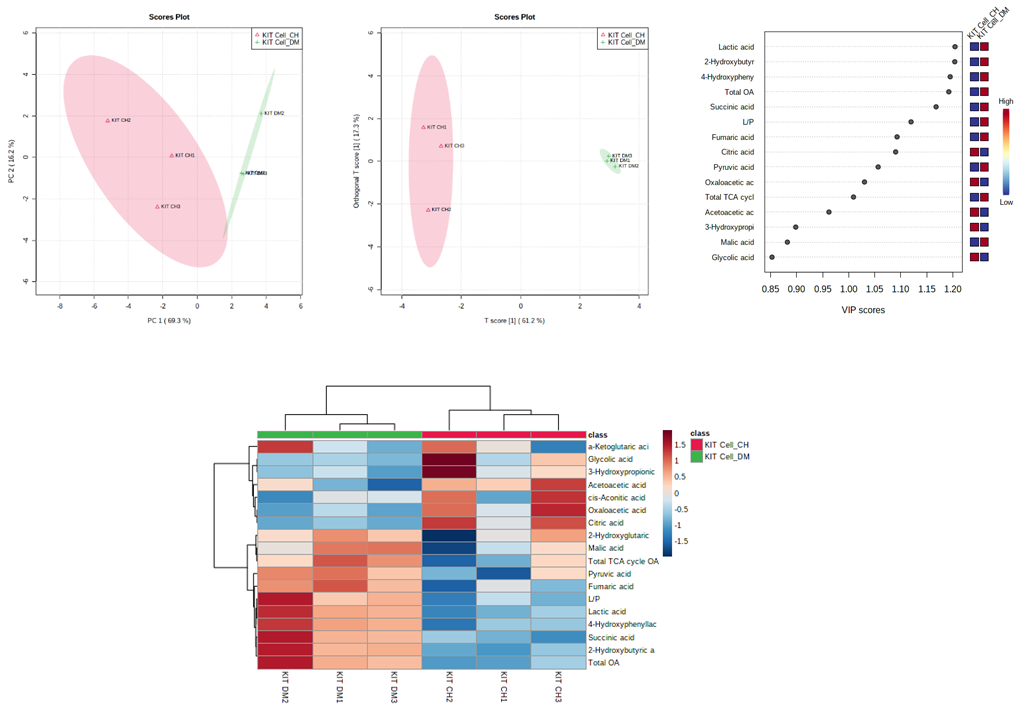


**Supplementary Figure 1.** PCA, OPLS-DA, VIP score, and heatmap analysis for CaCo2/KITENIN cell with DMSO and chrysophanol treatment.


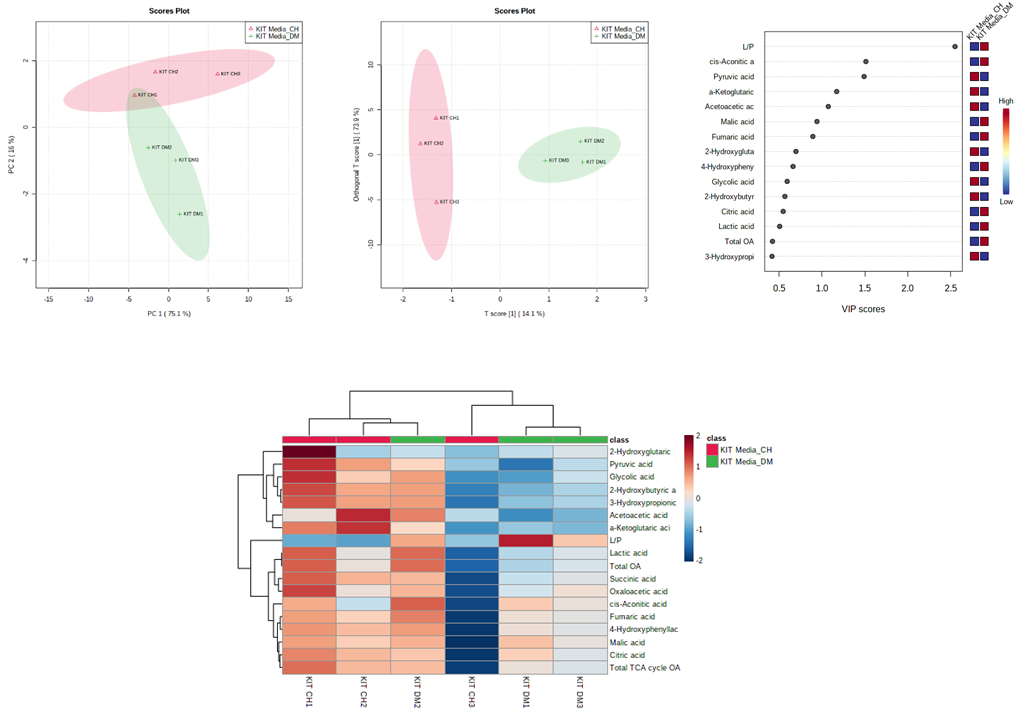


**Supplementary Figure 2.** PCA, OPLS-DA, VIP score, and heatmap analysis for CaCo2/KITENIN media samples treatment by DMSO and chrysophanol treatment.

**Supplementary Table 1.** qRT-PCR primers

| **Primers sequences** | | |
| --- | --- | --- |
| **Gene symbol** | **For (5'-3')** | **Rev (5'-3')** |
| GLUT1 | CTTTGTGGCCTTCTTTGAAGT | CCACACAGTTGCTCCACAT |
| HK2 | AAGGCTTCAAGGCATCTG | CCACAGGTCATCATAGTTCC |
| PKM1 | CGAGCCTCAAGTCACTCCAC | GTGAGCAGACCTGCCAGACT |
| PKM2 | ATTATTTGAGGAACTCCGCCGCCT | ATTCCGGGTCACAGCAATGATGG |
| LDHA | TGGCCTGTGCCATCAGTATC | TTCCAAGCCACGTAGGTCAA |
| β-catenin | AAAATGGCAGTGCGTTTAG | TTTGAAGGCAGTCTGTCGTA |
| c-Myc | AATGAAAAGGCCCCCAAGGTAGTTATCC | GTCGTTTCCGCAACAAGTCCTCTTC |
| Cyclin D1 | CCGTCCATGCGGAAGATC | GAAGACCTCCTCCTCGCACT |
| hnRNPI (PTBP1) | TCAGGCCTTCATCGAGATGCACA | TCTTGAGCTCCTTGTGGTTGGA |
| hnRNPA1 | GCTCACGGACTGTGTGGTAA | GGCCTTGCATTCATAGCTGC |
| hnRNPA2 | GGAGTGGAAGAGGAGGCAAC | CAGGTCCTCCTCCATACCCA |
| β-actin | ATTGTGAACTTTGGGGGATG | GATGAGATTGGCATGGCTTT |

**Supplementary Table 2.** Antibody information

| **Antibodies** | | |
| --- | --- | --- |
| Alpha tubulin (11h101) Rabbit mAb | Cell signaling | #2125 |
| GAPDH (D16H11) XP Rabbit mAb | Cell signaling | #5174 |
| Goat Anti-Rabbit IgG (H+L) Peroxidase Conjugated | Thermo scientific | 31460 |
| Goat Anti-Mouse IgG (H+L) Peroxidase Conjugated | Thermo scientific | NCI1430KR |
| PKM2 (D78A4) XP Rabbit mAB | Cell signaling | #4053 |
| GLUT1 (D3J3A) Rabbit mAB | Cell signaling | #12939 |
| LDHA (C4B5) Rabbit mAB | Cell signaling | #3582 |
| Beta-actin antibody | Cell signaling, #4967 | #4967 |
| Hexokinase II (C64G5) Rabbit mAb | Cell signaling, #2867 | #2867 |
| Anti-cyclin D1 Ab-3 mouse mAB | Merck, Kenilworth, NJ, USA | DCS-6 |
| PKM1 (D30G6) Rabbit mAB | Cell signaling | #7067 |
| Anti-VANGL1 | Atlas antibodies | HPA025235 |
| c-Myc (9E10) | Santa Cruz Biotech. | sc-40 |
| HER4/ErbB4 (111B2) Rabbit mAb | Cell signaling | #4795 |
| Vangl1 (E-3) | Santa Cruz Biotech. | sc-166844 |
